# Links of positive affect and stress to HbA1c: a prospective longitudinal study

**DOI:** 10.1007/s10865-023-00408-8

**Published:** 2023-04-18

**Authors:** Fiona S. Horner, Vicki S. Helgeson, Mary T. Korytkowski

**Affiliations:** 1 Department of Psychology, Carnegie Mellon University, 5000 Forbes Avenue, Pittsburgh, PA 15213 USA; 2 Center for Diabetes and Endocrinology, University of Pittsburgh Medical Center, Pittsburgh, USA

**Keywords:** Positive affect, Stress, HbA1c, Type 2 diabetes

## Abstract

While affect is linked to a number of diabetes outcomes, the specific role of positive affect (PA) in HbA1c remains unclear. The present study examined whether PA prospectively predicted lower HbA1c among adults with type 2 diabetes and whether this relation was moderated by stress. Participants were 123 adults (44.7% female; 60.2% White, 39.8% Black) recently diagnosed with type 2 diabetes. Perceived stress, diabetes-specific distress, and PA were assessed at baseline; HbA1c was assessed at baseline (T1), six months (T2), and five years (T3). PA was cross-sectionally associated with lower HbA1c at T1 and prospectively predicted lower HbA1c at T3. PA interacted with both measures of T1 stress to predict T1 HbA1c, and PA interacted with T3 perceived stress to predict T3 HbA1c. Interactions were consistent with stress buffering. Sensitivity analyses attentuated findings, but robust evidence remained for PA as a protective factor for blood glucose five years later and for a stress-buffering effect of PA on diabetes-specific distress. Findings suggest PA may be a clinically useful indicator among adults with type 2 diabetes and may be particularly important for those experiencing the greatest stress from their disease.

**Supplementary Information:**

The online version contains supplementary material available at 10.1007/s10865-023-00408-8.

## Introduction

Every year, 1.5 million adults in the United States are diagnosed with diabetes, with more than 90% of cases diagnosed as type 2 (US Department of Health & Human Services, 2020). People with diabetes face a complex disease management regimen that must be consistently maintained to prevent serious microvascular, neuropathic and macrovascular complications (Zheng et al., 2018). This challenging self-management regimen contributes to higher levels of daily stress, as people with diabetes work to maintain recommended diet and exercise plans, attend medical appointments, monitor glucose, and adhere to medication regimens.

Some people with diabetes struggle more with this transition to chronic disease management than others, and one factor that may influence an individual’s success in navigating this shift is positive affect (PA). PA, the experience of pleasant emotions, shows strong links to a number of positive health outcomes, including lower mortality risk and reduced risk of disease onset, with increased health behaviors serving as a major pathway through which these effects occur (Pressman & Cohen, 2005; Pressman et al., 2019). In the context of chronic illness, PA predicts reduced disease severity and slower disease progression in the domains of cancer, cardiovascular disease, and HIV/AIDS (Pressman et al., 2019). However, the role of PA in the context of diabetes remains understudied; instead research has focused on the robust detrimental effects of negative affect (NA) on disease onset, management, and outcomes (e.g., Gonzalez et al., 2007; Naicker et al., 2017; Skaff et al., 2009). While PA and NA do covary, they are conceptually and statistically distinct constructs: correlations tend to be modest, and each shows independent links to social, psychological, and physiological outcomes (Chida et al., 2008; Diener & Emmons, 1984; Steptoe et al., 2009). Thus, the emphasis on NA in diabetes gives only a partial picture as to how affect may impact adjustment to and management of this disease.

Some work has investigated PA and related constructs (e.g., resiliency, well-being) as protective factors in the context of diabetes (Robertson et al., 2012). For example, PA has been found to buffer the link between family history of diabetes and risk of developing the disease (Tsenkova et al., 2016), has been linked to increased physical activity among adults with diabetes (Spangler & Konen, 1993), and has been linked to reduced risk of all-cause mortality among people with diabetes (Moskowitz et al., 2008).

However, our understanding of the impact of PA on HbA1c, a measure of one’s average blood glucose over the prior two to three months, remains incomplete. Some cross-sectional and ecological momentary assessment studies have investigated the links between PA and HbA1c or blood glucose levels, but findings are mixed (Boylan et al., 2017; Papanas et al., 2010; Shapira et al., 2021; Skaff et al., 2009). Only a handful of studies have prospectively examined the effect of PA on HbA1c, but each of these studies is limited in some way. One study of adolescents with type 1 diabetes found PA to prospectively predicted lower HbA1c six months later, but NA was not statistically controlled (Lord et al., 2014). Thus, it is unclear whether these findings are due to high PA or the absence of NA, or if they would generalize to adults with diabetes or to people with type 2 diabetes. Another study of older women without diabetes found baseline PA to prospectively predicted lower HbA1c two years later, even after controlling for NA (Tsenkova et al., 2008). A nationally representative sample of adults in England who did not have diabetes found well-being (including constructs aside from affect, like autonomy and control) prospectively predicted lower HbA1c over a period of eight years, although PA was not directly assessed (Poole et al., 2020). While these findings are suggestive, it is unclear if results from persons without diabetes would generalize to those with diabetes. Thus, the goal of this study was to prospectively investigate the effect of PA on HbA1c among adults with type 2 diabetes.

Because it is unlikely that the role of PA in diabetes is uniform across everyone, our second study goal was to examine an important factor that might impact this relation—stress. Research has shown stress to moderate the relation between PA and health such that PA is most beneficial for individuals who report higher levels of stress (Pressman & Cohen, 2005). For example, one experiment on skin barrier disruption found PA to predict faster healing, but only among those assigned to a stress condition (Robles et al., 2009). The stress-buffering effect of PA on health has also been found in the context of immune function (Blevins et al., 2017) and mortality (Okely et al., 2017; see Pressman et al., 2019 for discussion). PA is more effective under conditions of high stress because it reduces stress appraisals and physiological stress reactivity, offers a “time out” from stress, and builds and mobilizes resources that can be used to address a stressor (Fredrickson, 2001; Pressman et al., 2019). These effects have the capacity to facilitate positive coping processes and, ultimately, better health.

To the extent that people with diabetes vary in the level of stress they experience—both in terms of general stress and stress specific to their disease—benefits of PA in relation to disease outcomes may differ across persons. Indeed, the intensity of one’s disease management regimen differs across people with type 2 diabetes, indicating one’s stress level likely varies as well. Little research has examined the interaction between stress and PA in the context of diabetes. In a notable exception, Moskowitz et al. (2008) examined whether PA buffered the effects of general subjective stress in adults with and without diabetes. They found that the association between PA and mortality was not moderated by stress among people with diabetes but was moderated by stress among older adults without diabetes. It is thus unclear if the stress-buffering effect of PA holds among people with diabetes. Further, it is unclear if diabetes-specific stress (e.g., burden of disease management, feeling that close others don’t understand this burden) is buffered by PA. The present research aims to answer these questions.

It should be noted that PA can occur across different spans of time. The present study involved secondary analysis of daily diary data, in which daily affect ratings across 14 days were averaged to form stable, dispositional measures of affect. This method avoids the recency and saliency biases associated with retrospective recall methods (Shiffman et al. 1997). Additionally, PA encompasses a range of emotions that vary in arousal level (e.g., calm versus excited). Here, we use a mid-arousal scale of general happiness as our measure of PA (Usala & Hertzog, 1989).

## The present study

The primary goal of the present study was to prospectively examine whether baseline PA predicted HbA1c among adults with type 2 diabetes over a five-year follow-up period. As PA may be particularly important for disease outcomes at earlier rather than later disease stages (Pressman & Cohen, 2005), we investigated the role of PA among adults who had recently been diagnosed with type 2 diabetes. Specifically, we examined whether baseline PA predicted HbA1c at baseline (T1), six months (T2), and five years (T3), independent of baseline NA. We hypothesized that PA would predict lower HbA1c at each timepoint.

Our second goal was to assess whether stress moderated these relations. We examined whether diabetes-specific distress or overall perceived stress interacted with baseline PA to predict HbA1c at each timepoint, hypothesizing that PA would be most protective for those reporting the highest stress. Finally, because Black and White people with diabetes may differ in the glycation of hemoglobin (Bergenstal et al., 2017) and the psychosocial processes influencing health (Lincoln et al., 2003), we took advantage of the fact that we had roughly equal numbers of Black and White participants and conducted exploratory analyses to examine potential interactions between race and PA in predicting the same set of outcomes.

## Method

The present study was part of a larger study on the role of the social environment in coping with diabetes. Follow up data were collected at six months (to identify short term changes in disease adjustment and relationships) and 5 years (to identify long term changes in glycemia and behaviors). Full study procedures are documented elsewhere (Zajdel & Helgeson, 2020), and full research materials are available at osf.io/bz8f6. Data and analysis code are available at osf.io/zg9d4. All analyses were conducted using R (v4.2.0; R Core Team, 2022).

## Procedure

Recruitment and study procedures were approved by the Carnegie Mellon University and University of Pittsburgh Institutional Review Boards. People who had been diagnosed with type 2 diabetes in the last 5 years were recruited in southwestern Pennsylvania from September 2012 to December 2017. Participants were recruited through hospital registries, mass advertising, and health fairs. Participants had to be married or cohabitating with a romantic partner who did not have diabetes to be eligible; while both couple members took part in the larger study, the present study used data only from participants with diabetes. Because type 2 diabetes disproportionately impacts Black communities, we aimed for our sample to be roughly half Black and half White.

Participants provided informed consent twice: once prior to baseline, which included consent for the six-month follow up assessment, and once prior to the five-year follow up assessment. At baseline (T1), participants completed an in-person interview during which they provided demographic and illness data along with measures of overall perceived stress, diabetes-specific distress, and HbA1c. Participants were provided with iPads to complete 14 daily diary surveys in which they reported their affect (i.e., PA, NA) at the end of each day. Daily diary compliance was high, with participants completing an average of 12.33 (*SD* = 1.66) out of 14 surveys. Six months (T2) and five years (T3) later, participants were invited by phone or email to participate in follow-ups interviews, during which they completed stress and HbA1c measures.

The COVID-19 pandemic began part way through collection of the five-year follow-up. As such, 22.8% of participants completed their five-year assessment remotely after stay-at-home orders began on March 16th, 2020.

## Participants

At baseline, 207 persons with diabetes participated in the study. Because seven participants did not complete the daily diary period during which PA was measured, the baseline sample size was *n* = 200. Of these, we retained 192 persons at T2 and 129 at T3. Reasons for non-participation at T3 included being unable to reach the participant (n = 26), passive refusal (n = 21), declining participation (n = 8), the participant passing away (n = 7), and other reasons (n = 2). Five participants did not have HbA1c data for T3 so were dropped from analysis. Thus, the final sample size for the present study was 123. We included those who had HbA1c data at all three time-points because this facilitates direct comparison across time. That is, if the sample size differed at each wave, findings would be confounded with these changes in sample composition. Of these participants, 44.7% were female and 55.3% were male; 77.2% were married and 22.8% were cohabitating with a romantic partner. Full demographic information is presented in Table 1.

**Table 1:** Baseline demographic and illness characteristics N = 123

	Percentages or *M* (*SD*)
Age	53.3 (11.6)
Gender	
Female	44.72%
Male	55.28%
Race	
White	60.16%
Black	39.84%
Length of marriage/cohabitation	19.88 (15.09)
Years since diagnosis	1.87 (1.72)
Insulin	22.00%
Education	
Some high school or high school graduate	30.10%
Some College	13.00%
2- or 4-year college graduate	43.10%
Postgraduate education	13.80%
Income	
Less than $30,000	20.32%
$30,000–$59,000	35.77%
$60,000–$89,000	22.76%
Greater than $90,000	21.14%

A comparison of participants lost to follow up or dropped from analyses to those who were retained in the present analyses revealed several differences in demographic variables. Those retained in the final sample were more likely to be White than Black (*p* = 0.02); whereas the baseline sample was 46.9% Black and 53.1% White, the final sample was 39.8% Black and 60.2% White. Those retained in the study also had lower baseline HbA1c (*M* = 6.89%) than those who were lost to follow-up (*M* = 7.63%,* t* = 2.81, *p* < 0.01). Additionally, those retained in the present analyses were more likely to have higher incomes (*p* = 0.006) and were marginally more likely to be married (*p* = 0.06) and more educated (*p* = 0.06). There were no differences in baseline PA, NA, perceived stress, or diabetes specific distress between the two groups.

### Measures

#### Affect

Affect was measured during the daily diary period using a series of four adjective rating scales that were largely based on the POMS (McNair et al., 1971; Usala & Hertzog, 1989). Participants rated each adjective on a scale from one to five, indicating how they had felt that day. PA was measured with the well-being subscale, including the items “happy,” “pleased,” and “cheerful” (reliability of change, *R*_*C*_ = 0.79; Bolger & Laurenceau, 2013). NA was measured with the depression (“depressed,” “sad,” “unhappy”; *R*_*C*_ = 0.79), anxiety (“nervous,” “anxious,” “relaxed”, *R*_*C*_ = 0.50) and anger (“angry,” “annoyed,” and “mad”; *R*_*C*_ = 0.81) subscales. Due to the poor reliability of the anxiety subscale, the item “relaxed” (reverse-scored) was dropped from analyses; the remaining items were correlated at *r* = 0.84, *p* < 0.001. Responses were averaged across the 14 daily surveys to achieve trait measures of PA, depression, anger, and anxiety. Finally, because the three NA measures were highly correlated (depression and anger, *r* = 0.81, *p* < 0.001; depression and anxiety, *r* = 0.82, *p* < 0.001, anger and anxiety, *r* = 0.71, *p* < 0.001), we created a composite NA index by averaging participants’ depression, anxiety, and anger scores.

#### Diabetes-specific distress

The Diabetes Distress Scale (Polonsky, 2005) was used to assess the extent to which participants felt distressed by their disease across four distinct domains: emotional burden (five items, e.g., “Feeling that diabetes is taking up too much of my mental and physical energy every day”), physician distress (four items, e.g., “Feeling that my doctor doesn’t take my concerns seriously enough”), interpersonal distress (three items, e.g., “Feeling that friends or family don’t appreciate how difficult living with diabetes can be”), and regimen distress (five items, e.g., “Not feeling confident in my day-to-day ability to manage diabetes”). Participants rated how much of a problem they perceived each item to be on a scale from 1 (no problem) to 6 (serious problem). The four subscales were combined into an overall index of diabetes distress which showed good reliability: T1 α = 0.89; T2 α = 0.91; T3 α = 0.91.

#### Perceived stress

A four-item abbreviated version of the Perceived Stress Scale (Cohen et al., 1983) was administered to assess overall stress (example item: “How often have you felt that you were unable to control the important things in your life?"). Participants responded on a scale from 1 (never) to 5 (very often). The combined scale showed sufficient reliability at each time point: T1 α = 0.81; T2 α = 0.79; T3 α = 0.74.

#### HbA1c

At T1 and T2, and for the majority (77.2%) of T3, study personnel measured HbA1c in-person with the DCA Vantage Analyzer. However, because T3 data collection was interrupted by the COVID-19 pandemic, a shift to remote measures was necessitated. As such, the remainder (22.8%) of T3 HbA1c measures were collected with CoreMedica HemaSpot-SE self-collection kits which participants mailed to a lab for processing. Lower HbA1c values indicate better disease management.

### Analysis plan

To examine potential differences between the DCA Vantage Analyzer (DCA) and the CoreMedica HemaSpot self-collection (CoreMedica) HbA1c tests, T3 HbA1c was compared across participants who used each measure. First, T3 HbA1c values were compared across measures with a t-test. Next, a change score was calculated by subtracting T2 HbA1c from T3 HbA1c, and the change scores were compared across the two measures. Finally, Pearson’s correlations of HbA1c scores across timepoints were computed separately for those who used DCA throughout the study and those who switched to CoreMedica at T3.

Demographic and illness covariates (age, sex, race, income, education, marital status, marriage length, years since diagnosis, and insulin use) were selected based on their statistical relations with PA and HbA1c. We opted for this method because we did not have an a priori rationale for why any specific variable would obscure the relation of PA to outcomes. In including covariates, our goal was to eliminate confounds with PA, rather than to control for every predictor of HbA1c. Each candidate covariate was tested for relations with baseline PA and HbA1c at T1, T2, and T3. Variables that were correlated with both PA and HbA1c (*p* < 0.05) were retained as covariates. T1 HbA1c was additionally included as a covariate in models predicting T2 or T3 HbA1c so that we could examine changes in HbA1c over time. Finally, because the COVID-19 pandemic interrupted data collection at T3, we created a dummy variable indicating whether T3 data collection occurred prior to or during the pandemic. This was included as a covariate in all models predicting T3 HbA1c. This variable captures both the onset of the pandemic (e.g., behavioral and psychological changes) as well as the switch to the CoreMedica HbA1c kits, as these changes occurred simultaneously and are inseparable.

PA and NA are conceptually distinct constructs; it is generally agreed that they are not simply opposite ends of a single continuum. However, statistically they overlap (Diener & Emmons, 1984). To identify the unique impact of PA on our outcomes of interest above and beyond the shared variance of PA and NA, we included the composite NA variable as a covariate in all models (Pressman et al., 2019).

Pearson’s correlations were computed for PA, NA, the stress measures, and HbA1c at each time point. Linear regression models were then fit with covariates added in a stepwise manner to identify how their inclusion impacted the predictive ability of PA on HbA1c at T1, T2, and T3. In step one, only PA, T1 HbA1c (T2 and T3 models only), and pandemic onset (T3 model only) were included. In step two, demographic/illness covariates and NA were added. To assess if the onset of the COVID-19 pandemic and the subsequent change in HbA1c test kits impacted the relation between T1 PA and T3 HbA1c, we tested if the timing of T3 measurement (pre- versus post-onset of the pandemic) interacted with PA to predict T3 HbA1c.

We then assessed whether T1 diabetes distress or perceived stress moderated the link between T1 PA and HbA1c. To do so, we examined interactions between T1 PA and T1 diabetes distress and between T1 PA and T1 perceived stress in separate models to predict HbA1c at each timepoint. Finally, we conducted exploratory analyses to examine if PA interacted with race to predict HbA1c at each timepoint. In each case, we included covariates in the stepwise manner outlined above. All regression coefficients were standardized to facilitate interpretation.

Because HbA1c was positively skewed at each timepoint, bias-corrected and accelerated bootstrapped 95% confidence intervals were computed from 5000 resamples for all models using the boot package in R (v1.3–27; Canty & Ripley, 2021). Thus, all standard errors (SEs) and confidence intervals (CIs) reported below are bootstrapped.

## Results

### Comparison of T3 HbA1c measures

At T3, 95 participants had their HbA1c measured with the DCA Vantage Analyzer and 28 used the CoreMedica Hema-Spot self-collection kits. There was no difference in T3 HbA1c across measures (*t* = − 0.83, *p* = 0.41). The comparison of change scores from T2 to T3 similarly found no difference based on type of test used at T3 (*t* = 0.34, *p* = 0.73). For participants who used the DCA test at all three timepoints, T1 and T3 HbA1c were correlated at *r* = 0.49 (*p* < 0.001) and T2 and T3 HbA1c were correlated at *r* = 0.51 (*p* < 0.001). For participants who switched to the CoreMedica tests at T3, T1 and T3 HbA1c were correlated at *r* = 0.57 (*p* < 0.01) and T2 and T3 HbA1c were correlated at *r* = 0.45 (*p* < 0.05). Thus, HbA1c correlations across timepoints did not substantially differ based on type of HbA1c test used at T3.

### Selection of covariates

Examination of the demographic and illness variables revealed that age was positively correlated with PA (*r* = 0.26, *p* < 0.01) and negatively correlated with HbA1c at all three time points (T1: *r* = − 0.31, *p* < 0.001; T2: *r* = − 0.26, *p* < 0.01; T3: *r* = − 0.27, *p* < 0.01). Additionally, race was associated with HbA1c such that Black participants had higher HbA1c than White participants at T1 (*r* = 0.31, *p* < 0.001) and T2 (*r* = 0.29, *p* < 0.01). While race was not related to PA, we controlled for race because research has shown HbA1c may differ systematically between White and Black persons (Bergenstal et al., 2017). None of the other background variables were related to both PA and HbA1c at any time of assessment. Thus in step one, models predicting T2 HbA1c controlled for T1 HbA1c, and models predicting T3 HbA1c controlled for T1 HbA1c and the binary COVID-19 variable. In step two, age, race, and NA were added to all models.

### Links of PA to HbA1c

Table 2 presents the descriptive statistics and Pearson’s correlation matrix for affect, stress (diabetes distress and perceived stress), and HbA1c. PA was negatively correlated with HbA1c at both T1 and T3. Of note, NA was not related to HbA1c at any time point.

**Table 2:** Descriptive statistics and correlations among predictor and outcome variables

		M (SD)	1	2	3	4	5	6
1	T1 Positive Affect	3.68 (.80)						
2	T1 Negative Affect	1.54 (.53)	− 0.62***					
3	T1 DM Distress	2.14 (.86)	− 0.53***	0.48***				
4	T1 Perceived Stress	2.19 (.86)	− 0.49***	0.56***	0.61***			
5	T1 HbA1c	6.89 (1.56)	− 0.27**	0.14	0.39***	0.33***		
6	T2 HbA1c	6.88 (1.54)	− 0.12	0.03	0.17†	0.13	0.77***	
7	T3 HbA1c	7.33 (1.79)	− 0.36***	0.08	0.22*	0.23**	0.52***	0.49***

^***^p < .001; **p < .01; *p < .05; †*p* < .1

Regression results and bootstrapped confidence intervals are presented in Table 3. In both the unadjusted and adjusted models, PA was associated with lower HbA1c at T1 and predicted lower HbA1c five years later at T3. PA did not predict lower HbA1c at T2. PA did not interact with pandemic onset to predict T3 HbA1c (β = 0.00, SE = 0.08, 95% CI = [− 0.21, 0.13]).

**Table 3:** HbA1c at each time point regressed on T1 positive affect; unadjusted and adjusted models. Bootstrapped 95% CIs and SEs*

		Step 1		Step 2	
		β (SE)	CI	β (SE)	CI
T1	PA	− **0.27 (0.10)**	**(**− **0.51, **− **0.10)**	− **0.23 (0.13)**	**(**− **0.53, **− **0.01)**
	NA	–	–	− 0.05 (0.12)	(− 0.34, 0.14)
	Race	–	–	**0.23 (0.09)**	**(0.08, 0.43)**
	Age	–	–	− 0.18 (0.09)	(− 0.39, − 0.03)
T2	PA	0.09 (0.06)	(− 0.03, 0.21)	0.07 (0.07)	(− 0.08, 0.20)
	NA	–	–	− 0.05 (0.07)	(− 0.20, 0.08)
	Race	–	–	0.05 (0.06)	(− 0.06, 0.17)
	Age	–	–	− 0.03 (0.05)	(− 0.14, 0.07)
	T1 HbA1c	**0.80 (0.13)**	**(0.51, 1.03)**	**0.77 (0.13)**	**(0.49, 1.00)**
T3	PA	− **0.24 (0.09)**	**(**− **0.43, **− **0.09)**	− **0.36 (0.12)**	**(**− **0.61, **− **0.16)**
	NA	–	–	− **0.22 (0.09)**	**(**− **0.41, **− **0.06)**
	Race	–	–	− 0.02 (0.09)	(− 0.18, 0.15)
	Age	–	–	− 0.10 (0.07)	(− 0.25, 0.03)
	T1 HbA1c	**0.47 (0.10)**	**(0.29, 0.68)**	**0.45 (0.10)**	**(0.26, 0.65)**
	Covid	− 0.06 (0.07)	(− 0.20, 0.08)	− 0.07 (0.07)	(− 0.21, 0.07)

^*^Bias corrected and accelerated bootstrapped SEs and CIs

Standardized beta coefficients

Predictors in which CIs exclude 0 are in bold

The link of T1 PA to change in HbA1c at T3 was further investigated to assess if the negative coefficient reflected PA predicting a reduction in HbA1c over time or PA predicting a smaller increase in HbA1c over time. For the full sample, HbA1c increased from T1 to T3 (*M* = 6.89% and 7.33%, respectively, *t* = 2.45, *p* < 0.05). However, among those who reported PA more than 1 standard deviation above the mean (*n* = 24), this change in HbA1c from T1 to T3 was non-significant (*M* = 6.43% and 6.56%, respectively, *t* = 0.49, *p* > 0.5). Thus, PA predicted smaller increases in HbA1c over time.^1^

### Interactions of PA with stress in predicting HbA1c

**Fig. 1:**
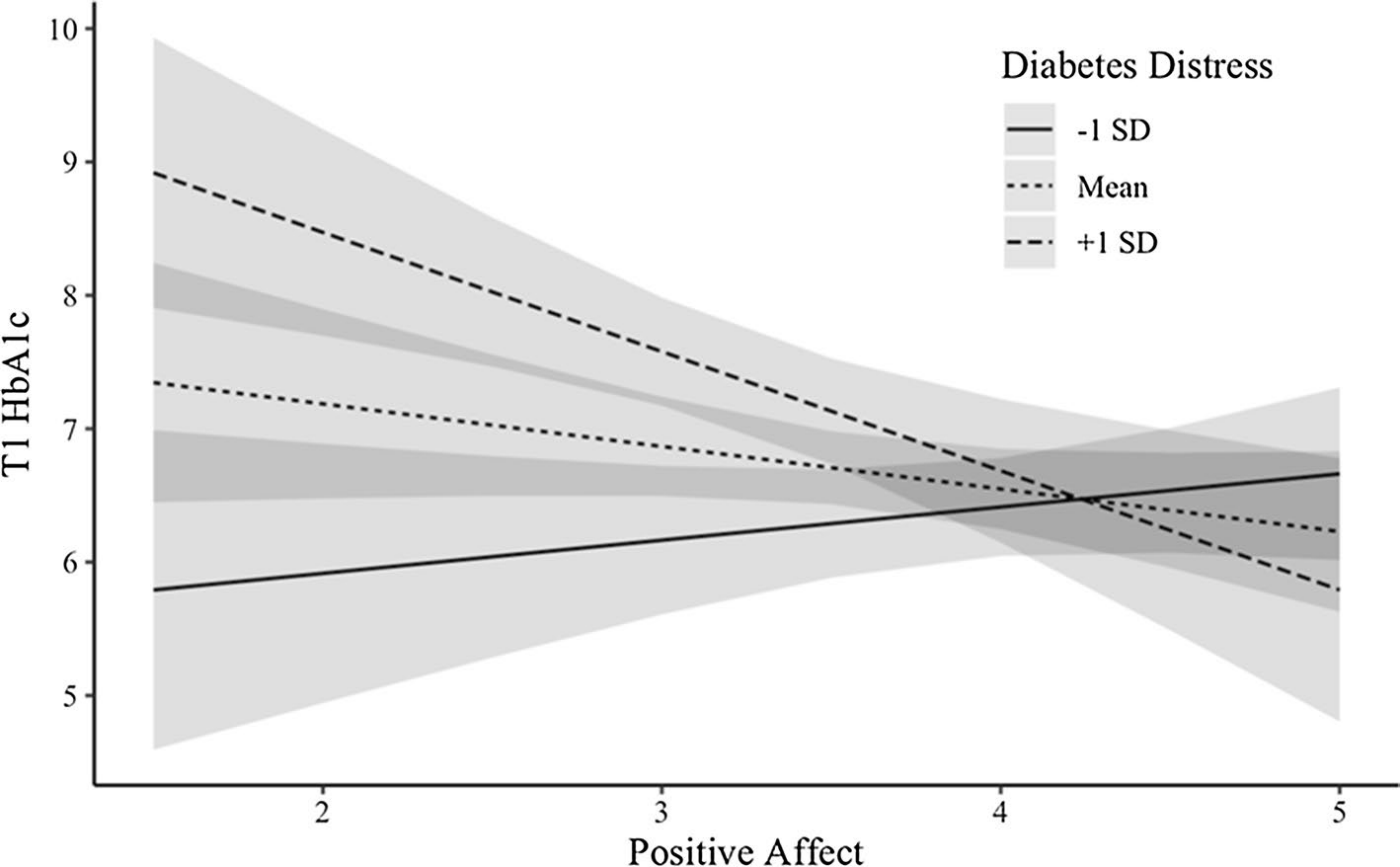
Diabetes distress moderates the link between PA and T1 HbA1c

**Fig. 2:**
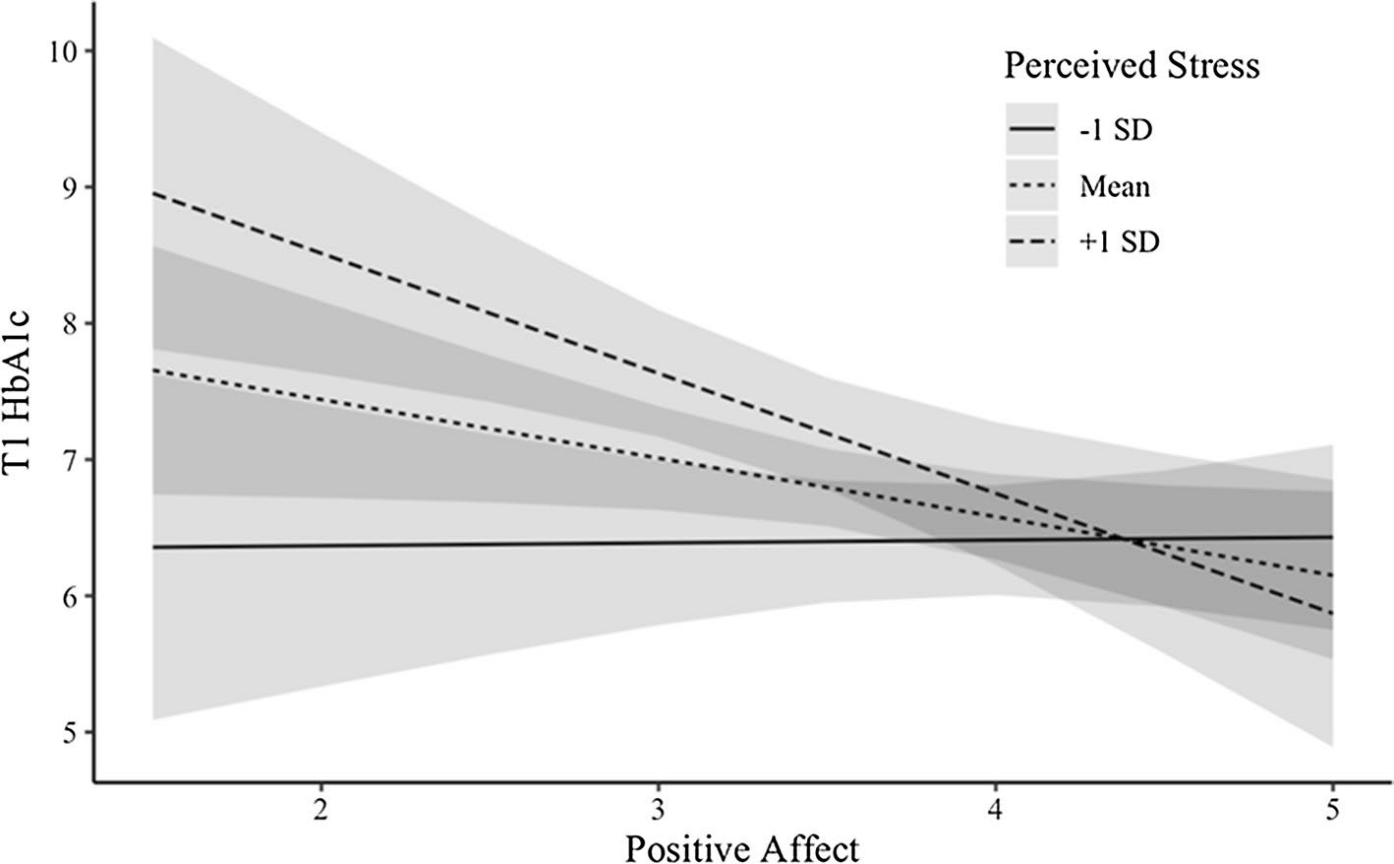
Perceived stress moderates the link between PA and T1 HbA1c

As shown in Table 4, there was an interaction between diabetes-specific distress and PA in the T1 model. Similarly, there was an interaction between overall perceived stress and PA in the T1 model. As predicted, both interactions were in the direction of PA being most protective at higher levels of stress (see Figs. 1 and 2). Simple slope analyses revealed that, for the interaction between PA and diabetes distress, the relation of PA to lower HbA1c was only significant when diabetes distress was high (i.e., 1 standard deviation above the mean or more; β = − 0.46, *p* < 0.01), but not when diabetes distress was at the mean (β = − 0.16, *p* > 0.1) or low (i.e., 1 standard deviation below the mean or more, β = 0.13, *p* > 0.1). Similarly, for the interaction between PA and perceived stress, the relation between PA and T1 HbA1c was significant when perceived stress was high (β = − 0.45, *p* < 0.01) or at mean levels (β = − 0.22, *p* < 0.05), but not when perceived stress was low (β = 0.01, *p* > 0.5).

**Table 4:** T1 HbA1c regressed on stress × PA interactions; adjusted and unadjusted models. Bootstrapped 95% CIs and SEs*

	Diabetes distress				Perceived stress				
	Step 1		Step 2		Step 1		Step 2		
	β (SE)	CI	β (SE)	CI	β (SE)	CI		β (SE)	CI
PA	− 0.11 (0.08)	(− 0.30, 0.03)	− 0.16 (0.11)	(− 0.41, 0.03)	− 0.14 (0.11)	(− 0.39, 0.04)		− 0.22 (0.13)	(− 0.52, − 0.00)
Stress	**0.23 (0.09)**	**(0.07, 0.42)**	**0.20 (0.08)**	**(0.05, 0.37)**	**0.23 (0.09)**	**(0.07, 0.43)**		**0.20 (0.10)**	**(0.03, 0.41)**
PA x Stress	− **0.29 (0.10)**	**(**− **0.50, **− **0.12)**	− **0.29 (0.09)**	**(**− **0.48, **− **0.12)**	− **0.18 (0.12)**	**(**− **0.44, 0.03)**		− **0.23 (0.11)**	**(**− **0.46, **− **0.02)**
NA	–	–	− 0.13 (0.11)	(− 0.43, 0.05)	–	–		− 0.19 (0.12)	(− 0.52, − 0.00)
Race	–	–	0.24 (0.08)	(0.09, 0.42)	–	–		0.24 (0.08)	(0.09, 0.43)
Age	–	–	− 0.10 (0.08)	(− 0.27, 0.04)	–	–		− 0.11 (0.09)	(− 0.30, 0.05)

^*^Bias corrected and accelerated bootstrapped SEs and CIs

Standardized beta coefficients

CIs excluding 0 are in bold

In the models predicting T2 and T3 HbA1c, there were no interactions between PA and stress (see Table S1 in supplement for complete results). Because this suggests that the impact of baseline stress did not last across follow-ups, we reasoned that stress measured at T2 and T3 may have greater implications for HbA1c at those time points. Thus, exploratory analyses were conducted to determine whether baseline PA interacted with T2 stress to predict T2 HbA1c and whether baseline PA interacted with T3 stress to predict T3 HbA1c.^2^ Results showed that at T2, neither diabetes specific stress (β = 0.05, SE = 0.11, 95% CI [− 0.14, 0.29]) nor perceived stress (β = 0.08, SE = 0.09, 95% CI [− 0.07, 0.31]) interacted with baseline PA to predict T2 HbA1c. Similarly, diabetes distress did not interact with baseline PA to predict T3 HbA1c (β = − 0.02, SE = 0.12, 95% CI [− 0.27, 0.20]). However, baseline PA interacted with T3 perceived stress to predict T3 HbA1c (β = − 0.15, SE = 0.10, 95% CI [− 0.38, − 0.01]). This interaction was such that PA predicted lower T3 HbA1c at all stress levels, but the link was strongest among those who reported highest stress.

Race did not moderate the relation between PA and HbA1c at any timepoint.

### Sensitivity analyses

To assess the robustness of findings, several sensitivity analyses were conducted. First, baseline CES-D (Radloff, 1977) was added to each model to assess if the inclusion of a formal depression measure—beyond the depressed affect in the NA scale—altered results. Second, due to concerns about multiple comparisons, models were rerun (without the CES-D) at the 99% confidence level. Finally, models were run with both the CES-D and 99% confidence level. Complete sensitivity results can be found in the supplement.

Both adding the CES-D and increasing the confidence to 99% resulted in the main effect of PA on T1 HbA1c falling to non-significance (with CES-D: β = − 0.19 SE = 0.14, 95% CI [− 0.52, 0.03] see supplementary Table S3; 99% confidence: β = − 0.23 (0.13), 99% CI [− 0.63, 0.04], see supplementary Table S5). Inclusion of the CES-D did not change any of the interaction findings (supplementary Table S4). However, increasing the confidence level to 99% resulted in the two perceived stress interactions falling to non-significance (T1: β = − 0.23 SE = 0.11, 99% CI [− 0.54, 0.04], see supplementary Table S6; β = − 0.15, SE = 0.10, 99% CI [− 0.49, 0.03]). Including both sensitivity tests in the same model did not result in any additional changes to findings (supplementary Tables S7 and S8). In all cases, the main effect of PA on T3 and the PA by diabetes distress interaction were unchanged.^3^

## Discussion

In this study, we proposed that PA would predict lower Hba1c over time among adults with type 2 diabetes due to the health-protective effects associated with PA. Results showed that PA was cross-sectionally associated with lower HbA1c at baseline and that PA predicted lower HbA1c five years later controlling for baseline HbA1c. This pattern of results was the same regardless of participant race. This research extends prior work on the protective effects of PA from other samples (i.e., adolescents with type 1 diabetes and adults without diabetes) to adults recently diagnosed with type 2 diabetes and supports the conclusion that PA predicts positive changes in HbA1c over time.

Of note, there was no zero-order correlation between baseline PA and HbA1c at six months, and baseline PA did not prospectively predict change in HbA1c six months later. At this point it is not clear why this was the case, or indeed why HbA1c at six months was unrelated to all of our psychosocial variables. More research is needed to understand these findings. Our results suggest that the processes through which PA influences HbA1c operate on the order of years rather than months.

There are multiple mechanisms through which PA may influence blood glucose levels. One likely pathway is through health-protective behaviors. Research has found PA to predict health behaviors like diet and exercise among the general public (Kushlev et al., 2020). A growing body of literature supports similar findings in the context of chronic illness (Bassett et al., 2019; Miles et al., 2018) although findings vary based on the specific health behavior in question and the PA arousal level (e.g., calm versus excited; Jones et al., 2021). To the extent that PA helps people with diabetes better adhere to diet, exercise, and medication regimens, it may subsequently lead to lower HbA1c levels. Relationships may similarly play a key role. PA is known to foster the accrual of social resources like increased social connectedness and improved relationship quality (Ramsey & Gentzler, 2015). These social resources, in turn, have strong links to improved health outcomes across a variety of contexts (Holt-Lunstad et al., 2010; Robles et al., 2014). Of note, the present sample was composed only of individuals in long-term romantic relationships. Given the specific role of romantic relationships in health (Robles et al., 2014), this pathway may be particularly important in this sample. Future research is needed to investigate these potential mediators and compare findings to those not in romantic relationships.

Importantly, the links between PA and HbA1c were moderated by stress. At baseline, both overall perceived stress and diabetes-specific distress interacted with PA to predict HbA1c. Further, exploratory analyses revealed that T3 perceived stress interacted with baseline PA to predict T3 HbA1c. All three interactions were such that PA was most protective at higher levels of stress, consistent with the stress-buffering hypothesis of PA and health (Pressman & Cohen, 2005). However, both perceived stress interactions were not robust to sensitivity analyses. This may be the result of low power from our somewhat small sample, or may indicate that, for those with diabetes, PA is more protective when it comes to diabetes distress versus general stress. These findings align with previous work that has found PA to “undo” the negative effects of stress, for example, by speeding up physiological recovery (e.g., heart rate) after a stressful task (Fredrickson, 2013). Relatedly, as PA both facilitates the accrual of social resources and helps people access those resources (e.g., by asking for help), it may lead people to more efficiently address stressors related to diabetes and subsequently engage in better disease management behaviors. Future research should thus investigate whether coping, particularly interpersonal coping, explains the observed stress buffering effects of PA.

While we here conceptualize PA as an independent variable that leads to positive behavioral and biological changes, it should be noted that affect has bidirectional links with other important factors that may be driving the observed effects. For example, one’s physical symptoms likely covary with PA and may predict disease outcomes, or people high in extraversion, which is marked by high PA, may adjust more quickly to a diabetes diagnosis. While the present study did not measure these variables, future research should explore the extent to which PA independently predicts blood glucose beyond these related factors.

In the clinical setting, our findings suggest that assessing dispositional PA at time of diagnosis may provide valuable information to the practitioner. By identifying patients with low PA who may be at risk for higher HbA1c, psychosocial and educational resources can be targeted towards those who may need them most. Further, these findings suggest that focusing on NA or depression may not fully characterize those who are at risk for poor adjustment after a diabetes diagnosis and indicate that PA interventions may be beneficial for patients at this stage, particularly those reporting high stress.

Strengths of the present study include the use of a daily PA measure to capture mean PA, a method that avoids the recency and saliency bias associated with retrospective trait affect measures (Shiffman et al., 1997). Further, the inclusion of NA as covariate allowed us to identify the unique predictive power of PA beyond that of the shared variance between PA and NA. Our sample was also diverse in terms of income and education, and with nearly 40% of the sample Black, we were able to confirm that our findings held across both Black and White people with diabetes. Finally, the present study took place over a lengthy follow up period, allowing us to investigate long-term links of PA to blood glucose.

We also note several study limitations. First, we had relatively high attrition, with only 123 of the original 207 participants included in these analyses. Attrition was also higher for those with higher HbA1c, lower income, and for Black participants. This differential attrition may reduce the generalizability of these findings and limit our power to detect moderation by race. Additionally, baseline HbA1c was measured just prior to the daily diary period during which affect was assessed. As HbA1c captures mean blood glucose across the previous three months, the baseline findings could be interpreted as HbA1c predicting PA. In averaging daily affect, our intention was to create a stable measure of PA representative of long-term affective trends, which should thus similarly represent a participant’s affect three months prior. While this method has been recommended in the affect literature (Merz & Roesch, 2011) it is possible that this two-week span was not fully representative of dispositional affect. However, the fact that this mean PA measure predicted HbA1c 5 years later indicates that its effects were, in fact, long lasting. Future research is recommended to replicate these findings using other measures of trait PA or by aggregating over longer diary periods. Additionally, the present research used only a single PA scale capturing happiness or well-being. Given the importance of arousal level in the link of PA to health (Pressman et al., 2019; Pressmen & Cohen, 2005), future research should investigate how findings may differ for positive emotions at varying arousal levels (calm vs. excitement). Ultimately, identifying the relevant dimensions and timescales of PA in the context of diabetes could lead to standardized measures of PA for clinical use.

Several limitations pertain to the follow up period of the present study. First, there were no assessments of HbA1c between 6 months and 5 years, so we were unable to examine how the predictive power of PA and stress evolved over this period. Collection of the five-year follow up data was also interrupted by the onset of the COVID-19 pandemic. This may have introduced substantial barriers to one’s diabetes care: participants may have delayed appointments in fear of contracting COVID-19, or loss of income and/or health insurance may have impacted one’s ability to adhere to medication and diet regimens. Alternatively, the reduction in social events may have increased control over one’s diet, for example by limiting meals out and facilitating meal planning, potentially assisting in disease management. Additionally, our measure of HbA1c changed to mail-in self test kits due to stay-at-home requirements. However, our investigation into whether these changes introduced bias into our results revealed no systematic differences.

In sum, we found robust evidence that PA predicted healthier HbA1c five years later, and weaker evidence for a cross-sectional link of PA to HbA1c. We also found that PA helped buffer against the negative effects of stress, especially diabetes-specific stress, on blood glucose levels. These findings position PA, independent of NA, as a potentially indicator of who may be at lower risk for poor diabetes outcomes. Identifying people with diabetes with low dispositional PA could allow for psychosocial resources to be targeted towards those who need them most.

## Supplementary Information

Below is the link to the electronic supplementary material.

Supplementary file1 (DOCX 57 kb)

## Data Availability

Study materials can be accessed at osf.io/bz8f6, and study data and analysis code can be accessed at osf.io/zg9d4.

## Footnotes

1 Although NA was not linked with T1 or T2 HbA1c, NA predicted lower HbA1c at T3. As this finding contradicts prior literature and NA did not show any zero-order correlations with HbA1c, we conducted additional analyses to assess whether this was a spurious suppression effect. We first tested whether removing NA from the model changed the effect of PA on T3 HbA1c; we then tested the reverse by removing PA. All other covariates were retained. Results showed that the effect of PA on HbA1c was robust to the inclusion of NA (= -0.23, SE = .08, 95% CI [-.41, -.08]). However, NA did not predict T3 HbA1c without PA in the model (= -.01, SE = .06, 95% CI [-.12, .11]). Thus, the T3 NA finding likely reflects a spurious suppression effect rather than a true relation.

2 Diabetes distress and HbA1c were correlated at T2 (*r* = .19 *p* < .05) and at T3 (*r* = .28, *p* < .01). Perceived stress and HbA1c were marginally correlated at T2 (*r* = .16, *p* < .10), and significantly correlated at T3 (*r* = .20, *p* < .05).

3 Baseline CESD was uncorrelated with HbA1c at all timepoints and did not predict T1 or T3 HbA1c. However, at T2, baseline CESD predicted lower HbA1c (= -.20 (.09), 95% bootstrapped CI [-.40, -.04]). This finding is counter to prior literature and theory, and at this point cannot be explained. When the CES-D models were rerun at the 99% confidence level, CESD did not predict HbA1c at any timepoint.
