# Supplementary material for: Links of positive affect and stress to HbA1c: a prospective longitudinal study
Source: J Behav Med. 2023 Apr 18;46(5):849–59. doi: 10.1007/s10865-023-00408-8 (PMC10111289; doi:10.1007/s10865-023-00408-8)
Supplement: Supplementary file 1 — Supplementary file1 (DOCX 57 kb) [file 10865_2023_408_MOESM1_ESM.docx]

Links of Positive Affect and Stress to HbA1c: A Prospective Longitudinal Study

Supplementary Materials

**Contents**

1. **T2 and T3 PA by T1 Stress interactions** 2

Table S1: T2 and T3 HbA1c regressed on stress x PA interactions. 2

1. **Sensitivity analyses: CES-D included as covariate**

Table S2: Correlation matrix with primary study variables and CES-D. 3

Table S3: Primary regression models with CES-D added as covariate. 4

Table S4: PA x T1 Stress interaction models with CES-D included as covariate. 5

1. **Sensitivity analyses: 99% confidence level**

Table S5: Primary regression models, 99% confidence level. 6

Table S6: PA x T1 Stress interaction models, 99% confidence level. 7

1. **Sensitivity analyses: CES-D included and 99% confidence level**

Table S7: Primary regression models with CES-D, 99% confidence level. 8

Table S8: PA x T1 Stress interaction models with CES-D, 99% confidence level. 9

1. **T2 and T3 PA by Stress interactions**

Table S1. T2 and T3 HbA1c regressed on stress x PA interactions.

Bootstrapped 95% CIs and SEs.

|  |  | Diabetes Distress | | | | Perceived Stress | | | | |
| --- | --- | --- | --- | --- | --- | --- | --- | --- | --- | --- |
|  |  | Step 1 | | Step 2 | | Step 1 | | Step 2 | | |
|  |  | **β (SE)** | **CI** | **β (SE)** | **CI** | **β (SE)** | **CI** | | **β (SE)** | **CI** |
| T2 | PA | 0.04 (0.07) | (-0.10, 0.16) | 0.04 (0.08) | (-0.12, 0.19) | 0.04 (0.07) | (-0.10, 0.17) | | 0.07 (0.08) | (-0.09, 0.23) |
|  | Stress | -0.11 (0.07) | (-0.28, 0.00) | -0.13 (0.07) | (-0.30, 0.00) | -0.11 (0.08) | (-0.26, 0.04) | | -0.15 (0.08) | (-0.31, -0.00) |
|  | PA * Stress | 0.12 (0.10) | (-0.05, 0.35) | 0.12 (0.10) | (-0.06, 0.35) | 0.14 (0.10) | (-0.04, 0.36) | | 0.15 (0.11) | (-0.05, 0.37) |
|  | NA | -- | -- | 0.00 (0.07) | (-0.12, 0.14) | -- | -- | | 0.06 (0.07) | (-0.08, 0.21) |
|  | Race | -- | -- | 0.03 (0.06) | (-0.08, 0.15) | -- | -- | | 0.03 (0.06) | (-0.07, 0.16) |
|  | Age | -- | -- | -0.06 (0.06) | (-0.19, 0.06) | -- | -- | | -0.08 (0.06) | (-0.20, 0.03) |
|  | T1 HbA1c | **0.88 (0.12)** | **(0.58, 1.08)** | **0.85 (0.12)** | **(0.55, 1.06)** | **0.85 (0.12)** | **(0.56, 1.05)** | | **0.83 (0.12)** | **(0.55, 1.03)** |
|  |  |  |  |  |  |  |  | |  |  |
| T3 | PA | **-0.31 (0.10)** | **(-0.53, -0.13)** | **-0.40 (0.12)** | **(-0.66, -0.18)** | **-0.27 (0.09)** | **(-0.47, -0.10)** | | **-0.39 (0.12)** | **(-0.64, -0.17)** |
|  | Stress | -0.15 (0.09) | (-0.32, 0.03) | -0.12 (0.08) | (-0.29, 0.05) | -0.07 (0.09) | (-0.23, 0.10) | | 0.00 (0.09) | (-0.17, 0.21) |
|  | PA x Stress | -0.02 (0.09) | (-0.22, 0.13) | -0.03 (0.09) | (-0.22, 0.13) | -0.11 (0.10) | (-0.32, 0.06) | | -0.16 (0.10) | (-0.38, 0.03) |
|  | NA | -- | -- | **-0.19 (0.09)** | **(-0.38, -0.02)** | -- | -- | | **-0.27 (0.10)** | **(-0.50, -0.09)** |
|  | Race | -- | -- | -0.02 (0.09) | (-0.18, 0.16) | -- | -- | | 0.01 (0.09) | (-0.16, 0.20) |
|  | Age | -- | -- | -0.11 (0.07) | (-0.27, 0.02) | -- | -- | | -0.08 (0.08) | (-0.23, 0.07) |
|  | T1 HbA1c | **0.50 (0.10)** | **(0.33, 0.71)** | **0.47 (0.10)** | **(0.27, 0.67)** | **0.46 (0.11)** | **(0.26, 0.69)** | | **0.41 (0.11)** | **(0.20, 0.62)** |
|  | Covid | -0.08 (0.08) | (-0.22, 0.08) | -0.08 (0.08) | (-0.23, 0.08) | -0.08 (0.08) | (-0.22, 0.08) | | -0.09 (0.08) | (-0.24, 0.06) |

1. **Sensitivity analyses: CES-D included as covariate**

Table S2.

Correlation matrix with primary study variables and CES-D.

|  |  | **1** | **2** | **3** | **4** | **5** | **6** | **7** |
| --- | --- | --- | --- | --- | --- | --- | --- | --- |
| 1 | T1 CES-D |  |  |  |  |  |  |  |
| 2 | T1 PA | -0.55*** |  |  |  |  |  |  |
| 3 | T1 NA | 0.62*** | -0.62*** |  |  |  |  |  |
| 4 | T1 Diabetes Distress | 0.60*** | -0.53*** | 0.48*** |  |  |  |  |
| 5 | T1 Perceived Stress | 0.74*** | -0.49*** | 0.56*** | 0.61*** |  |  |  |
| 6 | T1 HbA1c | 0.29** | -0.27** | 0.14 | 0.39*** | 0.33*** |  |  |
| 7 | T2 HbA1c | 0.07 | -0.12 | 0.03 | 0.17† | 0.13 | 0.77*** |  |
| 8 | T3 HbA1c | 0.23** | -0.36*** | 0.08 | 0.22* | 0.23** | 0.52*** | 0.49*** |

Table S3.

Primary regression models with CES-D added as covariate.

Bootstrapped 95% CIs and SEs*

|  |  | **β (SE)** | **CI** |
| --- | --- | --- | --- |
| T1 | PA | -0.19 (0.14) | (-0.52, 0.03) |
|  | NA | -0.12 (0.12) | (-0.41, 0.07) |
|  | Race | **0.22 (0.09)** | **(0.07, 0.41)** |
|  | Age | **-0.18 (0.09)** | **(-0.39, -0.03)** |
|  | CES-D | 0.17 (0.14) | (-0.15, 0.42) |
|  |  |  |  |
| T2 | PA | 0.02 (0.07) | (-0.11, 0.15) |
|  | NA | 0.05 (0.07) | (-0.10, 0.18) |
|  | Race | 0.06 (0.06) | (-0.05, 0.19) |
|  | Age | -0.03 (0.05) | (-0.14, 0.07) |
|  | T1 HbA1c | **0.80 (0.12)** | **(0.50, 1.01)** |
|  | CES-D | **-0.20 (0.09)** | **(-0.40, -0.04)** |
|  |  |  |  |
| T3 | PA | **-0.36 (0.11)** | **(-0.60, -0.16)** |
|  | NA | **-0.23 (0.11)** | **(-0.48, -0.03)** |
|  | Race | -0.03 (0.08) | (-0.20, 0.14) |
|  | Age | -0.10 (0.07) | (-0.25, 0.03) |
|  | T1 HbA1c | **0.44 (0.10)** | **(0.25, 0.65)** |
|  | Covid | -0.07 (0.08) | (-0.21, 0.08) |
|  | CES-D | 0.03 (0.13) | (-0.19, 0.31) |

*Bias corrected and accelerated bootstrapped SEs and CIs.

Standardized beta coefficients

Predictors in which CIs exclude 0 are in bold.

Table S4.

PA x T1 Stress interaction models with CES-D included as covariate.

Bootstrapped 95% CIs and SEs.*

|  |  | Diabetes Distress | | Perceived Stress | |
| --- | --- | --- | --- | --- | --- |
|  |  | **β (SE)** | **CI** | **β (SE)** | **CI** |
| T1 | PA | -0.17 (0.12) | (-0.43, 0.03) | -0.22 (0.14) | (-0.56, 0.00) |
|  | Stress | **0.21 (0.10)** | **(0.03, 0.44)** | 0.22 (0.12) | (-0.03, 0.46) |
|  | PA x Stress | **-0.30 (0.10)** | **(-0.49, -0.12)** | **-0.24 (0.12)** | **(-0.48, -0.01)** |
|  | NA | -0.12 (0.11) | (-0.39, 0.05) | -0.19 (0.12) | (-0.50, 0.01) |
|  | Race | **0.24 (0.08)** | **(0.09, 0.43)** | **0.24 (0.09)** | **(0.09, 0.43)** |
|  | Age | -0.10 (0.08) | (-0.29, 0.05) | -0.11 (0.09) | (-0.31, 0.05) |
|  | CES-D | -0.02 (0.15) | (-0.37, 0.24) | -0.03 (0.18) | (-0.41, 0.30) |
|  |  |  |  |  |  |
| T2 | PA | 0.02 (0.08) | (-0.14, 0.17) | 0.05 (0.08) | (-0.11, 0.20) |
|  | Stress | -0.09 (0.07) | (-0.26, 0.03) | -0.09 (0.10) | (-0.30, 0.10) |
|  | PA x Stress | 0.10 (0.10) | (-0.08, 0.32) | 0.13 (0.11) | (-0.06, 0.36) |
|  | NA | 0.05 (0.07) | (-0.09, 0.19) | 0.08 (0.08) | (-0.06, 0.24) |
|  | Race | 0.04 (0.06) | (-0.07, 0.16) | 0.04 (0.06) | (-0.07, 0.17) |
|  | Age | -0.07 (0.06) | (-0.21, 0.05) | -0.08 (0.06) | (-0.21, 0.03) |
|  | T1 HbA1c | **0.85 (0.12)** | **(0.55, 1.06)** | **0.83 (0.12)** | **(0.54, 1.02)** |
|  | CES-D | -0.14 (0.08) | (-0.29, 0.02) | -0.12 (0.11) | (-0.33, 0.08) |
|  |  |  |  |  |  |
| T3 | PA | **-0.39 (0.12)** | **(-0.64, -0.18)** | **-0.39 (0.12)** | **(-0.65, -0.18)** |
|  | Stress | -0.14 (0.09) | (-0.32, 0.04) | 0.02 (0.12) | (-0.20, 0.26) |
|  | PA x Stress | -0.02 (0.08) | (-0.20, 0.13) | -0.16 (0.10) | (-0.38, 0.02) |
|  | NA | **-0.22 (0.11)** | **(-0.45, -0.02)** | **-0.26 (0.11)** | **(-0.50, -0.06)** |
|  | Race | -0.03 (0.09) | (-0.20, 0.14) | 0.01 (0.09) | (-0.16, 0.20) |
|  | Age | -0.11 (0.08) | (-0.26, 0.03) | -0.08 (0.08) | (-0.24, 0.07) |
|  | T1 HbA1c | **0.47 (0.11)** | **(0.27, 0.68)** | **0.41 (0.11)** | **(0.20, 0.63)** |
|  | Covid | -0.08 (0.08) | (-0.23, 0.08) | -0.09 (0.08) | (-0.25, 0.06) |
|  | CES-D | 0.07 (0.13) | (-0.15, 0.36) | -0.03 (0.14) | (-0.30, 0.25) |

*Bias corrected and accelerated bootstrapped SEs and CIs.

Standardized beta coefficients

Predictors in which CIs exclude 0 are in bold.

1. **Sensitivity analyses: 99% confidence level**

Table S5.

Primary regression models, 99% confidence level.

Bootstrapped CIs and SEs.*

|  |  | Step 1 | | Step 2 | |
| --- | --- | --- | --- | --- | --- |
|  |  | **β (SE)** | **CI** | **β (SE)** | **CI** |
| T1 | PA | **-0.27 (0.10)** | **(-0.59, -0.06)** | -0.23 (0.13) | (-0.63, 0.04) |
|  | NA | -- | -- | -0.05 (0.12) | (-0.43, 0.19) |
|  | Race | -- | -- | **0.23 (0.09)** | **(0.03, 0.49)** |
|  | Age | -- | -- | -0.18 (0.09) | (-0.46, 0.01) |
|  |  |  |  |  |  |
| T2 | PA | 0.09 (0.06) | (-0.06, 0.25) | 0.07 (0.07) | (-0.12, 0.25) |
|  | NA | -- | -- | -0.05 (0.07) | (-0.28, 0.13) |
|  | Race | -- | -- | 0.05 (0.06) | (-0.09, 0.21) |
|  | Age | -- | -- | -0.03 (0.05) | (-0.19, 0.10) |
|  | T1 HbA1c | **0.80 (0.13)** | **(0.40, 1.09)** | **0.77 (0.13)** | **(0.37, 1.06)** |
|  |  |  |  |  |  |
| T3 | PA | **-0.24 (0.09)** | **(-0.50, -0.04)** | **-0.36 (0.12)** | **(-0.69, -0.10)** |
|  | NA | -- | -- | **-0.22 (0.09)** | **(-0.49, -0.01)** |
|  | Race | -- | -- | -0.02 (0.09) | (-0.24, 0.22) |
|  | Age | -- | -- | -0.10 (0.07) | (-0.30, 0.08) |
|  | T1 HbA1c | **0.47 (0.10)** | **(0.25, 0.77)** | **0.45 (0.10)** | **(0.21, 0.72)** |
|  | COVID | -0.06 (0.07) | (-0.24, 0.13) | -0.07 (0.07) | (-0.27, 0.13) |

*Bias corrected and accelerated bootstrapped SEs and CIs.

Standardized beta coefficients

Predictors in which CIs exclude 0 are in bold.

Table S6.

PA x T1 Stress interaction models, 99% confidence level.

Bootstrapped CIs and SEs.*

|  |  | **Diabetes Distress** | | | | **Perceived Stress** | | | |
| --- | --- | --- | --- | --- | --- | --- | --- | --- | --- |
|  |  | Step 1 | | Step 2 | | Step 1 | | Step 2 | |
|  |  | **β (SE)** | **CI** | **β (SE)** | **CI** | **β (SE)** | **CI** | **β (SE)** | **CI** |
| T1 | PA | **-0.11 (0.08)** | **(-0.37, 0.07)** | -0.26 (0.17) | (-0.79, 0.12) | -0.14 (0.11) | (-0.50, 0.09) | -0.22 (0.13) | (-0.63, 0.05) |
|  | Stress | **0.23 (0.09)** | **(0.02, 0.48)** | **0.32 (0.13)** | **(0.01, 0.70)** | **0.23 (0.09)** | **(0.02, 0.49)** | 0.20 (0.10) | (-0.03, 0.47) |
|  | PA * Stress | **-0.29 (0.10)** | **(-0.60, -0.07)** | **-0.46 (0.14)** | **(-0.86, -0.11)** | -0.18 (0.12) | (-0.53, 0.10) | -0.23 (0.11) | (-0.54, 0.04) |
|  | NA | -- | -- | -0.21 (0.18) | (-0.87, 0.15) | -- | -- | -0.19 (0.12) | (-0.62, 0.06) |
|  | Race | -- | -- | **0.37 (0.13)** | **(0.08, 0.74)** | -- | -- | **0.24 (0.08)** | **(0.04, 0.49)** |
|  | Age | -- | -- | -0.16 (0.12) | (-0.52, 0.14) | -- | -- | -0.11 (0.09) | (-0.38, 0.10) |
|  |  |  |  |  |  |  |  |  |  |
| T2 | PA | 0.04 (0.07) | (-0.17, 0.20) | 0.04 (0.08) | (-0.18, 0.25) | 0.04 (0.07) | (-0.14, 0.22) | 0.07 (0.08) | (-0.15, 0.28) |
|  | Stress | -0.11 (0.07) | (-0.35, 0.03) | -0.13 (0.07) | (-0.36, 0.03) | -0.11 (0.08) | (-0.31, 0.08) | -0.15 (0.08) | (-0.36, 0.05) |
|  | PA * Stress | 0.12 (0.10) | (-0.10, 0.42) | 0.12 (0.10) | (-0.10, 0.42) | 0.14 (0.10) | (-0.10, 0.42) | 0.15 (0.11) | (-0.11, 0.45) |
|  | NA | -- | -- | 0.00 (0.07) | (-0.16, 0.19) | -- | -- | 0.06 (0.07) | (-0.13, 0.27) |
|  | Race | -- | -- | 0.03 (0.06) | (-0.11, 0.20) | -- | -- | 0.03 (0.06) | (-0.11, 0.20) |
|  | Age | -- | -- | -0.06 (0.06) | (-0.25, 0.09) | -- | -- | -0.08 (0.06) | (-0.25, 0.06) |
|  | T1 HbA1c | **0.88 (0.12)** | **(0.48, 1.15)** | **0.85 (0.12)** | **(0.44, 1.13)** | **0.85 (0.12)** | **(0.45, 1.11)** | **0.83 (0.12)** | **(0.43, 1.08)** |
|  |  |  |  |  |  |  |  |  |  |
| T3 | PA | **-0.31 (0.10)** | **(-0.60, -0.07)** | **-0.40 (0.12)** | **(-0.74, -0.11)** | **-0.27 (0.09)** | **(-0.54, -0.05)** | **-0.39 (0.12)** | **(-0.72, -0.11)** |
|  | Stress | -0.15 (0.09) | (-0.38, 0.09) | -0.12 (0.08) | (-0.35, 0.11) | -0.07 (0.09) | (-0.29, 0.16) | 0.00 (0.09) | (-0.22, 0.28) |
|  | PA * Stress | -0.02 (0.09) | (-0.30, 0.18) | -0.03 (0.09) | (-0.31, 0.17) | -0.11 (0.10) | (-0.40, 0.10) | -0.16 (0.10) | (-0.47, 0.08) |
|  | NA | -- | -- | -0.19 (0.09) | (-0.45, 0.04) | -- | -- | **-0.27 (0.10)** | **(-0.58, -0.04)** |
|  | Race | -- | -- | -0.02 (0.09) | (-0.23, 0.23) | -- | -- | 0.01 (0.09) | (-0.20, 0.27) |
|  | Age | -- | -- | -0.11 (0.07) | (-0.31, 0.06) | -- | -- | -0.08 (0.08) | (-0.28, 0.11) |
|  | T1 HbA1c | **0.50 (0.10)** | **(0.27, 0.81)** | **0.47 (0.10)** | **(0.21, 0.74)** | **0.46 (0.11)** | **(0.21, 0.77)** | **0.41 (0.11)** | **(0.14, 0.70)** |
|  | Covid | -0.08 (0.08) | (-0.27, 0.14) | -0.08 (0.08) | (-0.28, 0.14) | -0.08 (0.08) | (-0.26, 0.13) | -0.09 (0.08) | (-0.29, 0.13) |

*Bias corrected and accelerated bootstrapped SEs and CIs.

Standardized beta coefficients

Predictors in which CIs exclude 0 are in bold.

1. **Sensitivity analyses: CES-D included and 99% confidence level**

Table S7.

Primary regression models with CES-D, 99% confidence level

Bootstrapped CIs and SEs.*

|  |  | **β (SE)** | **CI** |
| --- | --- | --- | --- |
| T1 | PA | -0.19 (0.14) | (-0.63, 0.08) |
|  | NA | -0.12 (0.12) | (-0.50, 0.14) |
|  | Race | **0.22 (0.09)** | **(0.02, 0.49)** |
|  | Age | -0.16 (0.09) | (-0.44, 0.06) |
|  | CES-D | 0.17 (0.14) | (-0.26, 0.49) |
|  |  |  |  |
| T2 | PA | 0.02 (0.07) | (-0.16, 0.20) |
|  | NA | 0.05 (0.07) | (-0.15, 0.23) |
|  | Race | 0.06 (0.06) | (-0.08, 0.23) |
|  | Age | -0.06 (0.06) | (-0.23, 0.09) |
|  | T1 HbA1c | **0.80 (0.12)** | **(0.39, 1.07)** |
|  | CES-D | -0.20 (0.09) | (-0.47, 0.01) |
|  |  |  |  |
| T3 | PA | **-0.36 (0.11)** | **(-0.66, -0.10)** |
|  | NA | -0.23 (0.11) | (-0.57, 0.02) |
|  | Race | -0.03 (0.08) | (-0.25, 0.20) |
|  | Age | -0.10 (0.07) | (-0.30, 0.09) |
|  | T1 HbA1c | **0.44 (0.10)** | **(0.20, 0.72)** |
|  | COVID | -0.07 (0.08) | (-0.26, 0.13) |
|  | CES-D | 0.03 (0.13) | (-0.24, 0.41) |

*Bias corrected and accelerated bootstrapped SEs and CIs.

Standardized beta coefficients

Predictors in which CIs exclude 0 are in bold.

Table S8.

PA x T1 Stress interaction models with CES-D, 99% confidence level.

Bootstrapped CIs and SEs.*

|  | **Diabetes Distress** | | **Perceived Stress** | |
| --- | --- | --- | --- | --- |
|  | **β (SE)** | **CI** | **β (SE)** | **CI** |
| PA | -0.26 (0.18) | (-0.81, 0.14) | -0.22 (0.14) | (-0.66, 0.06) |
| Stress | 0.33 (0.16) | (-0.03, 0.81) | 0.22 (0.12) | (-0.11, 0.55) |
| PA * Stress | **-0.46 (0.15)** | **(-0.88, -0.11)** | -0.24 (0.12) | (-0.56, 0.04) |
| NA | -0.19 (0.17) | (-0.76, 0.17) | -0.19 (0.12) | (-0.61, 0.07) |
| Race | **0.37 (0.13)** | **(0.08, 0.78)** | **0.24 (0.09)** | **(0.05, 0.51)** |
| Age | -0.16 (0.13) | (-0.54, 0.16) | -0.11 (0.09) | (-0.39, 0.10) |
| CES-D | -0.03 (0.24) | (-0.77, 0.48) | -0.03 (0.18) | (-0.54, 0.40) |
|  |  |  |  |  |
| PA | 0.02 (0.08) | (-0.19, 0.22) | 0.05 (0.08) | (-0.16, 0.25) |
| Stress | -0.09 (0.07) | (-0.32, 0.07) | -0.09 (0.10) | (-0.35, 0.16) |
| PA * Stress | 0.10 (0.10) | (-0.13, 0.40) | 0.13 (0.11) | (-0.13, 0.42) |
| NA | 0.05 (0.07) | (-0.14, 0.24) | 0.08 (0.08) | (-0.11, 0.31) |
| Race | 0.04 (0.06) | (-0.11, 0.21) | 0.04 (0.06) | (-0.11, 0.21) |
| Age | -0.07 (0.06) | (-0.26, 0.08) | -0.08 (0.06) | (-0.25, 0.06) |
| T1 HbA1c | **0.85 (0.12)** | **(0.44, 1.12)** | **0.83 (0.12)** | **(0.43, 1.08)** |
| CES-D | -0.14 (0.08) | (-0.33, 0.08) | -0.12 (0.11) | (-0.40, 0.16) |
|  |  |  |  |  |
| PA | **-0.39 (0.12)** | **(-0.71, -0.12)** | **-0.39 (0.12)** | **(-0.72, -0.12)** |
| Stress | -0.14 (0.09) | (-0.39, 0.11) | 0.02 (0.12) | (-0.26, 0.36) |
| PA * Stress | -0.02 (0.08) | (-0.28, 0.18) | -0.16 (0.10) | (-0.47, 0.07) |
| NA | -0.22 (0.11) | (-0.52, 0.04) | -0.26 (0.11) | (-0.57, 0.00) |
| Race | -0.03 (0.09) | (-0.25, 0.21) | 0.01 (0.09) | (-0.20, 0.27) |
| Age | -0.11 (0.08) | (-0.31, 0.08) | -0.08 (0.08) | (-0.28, 0.12) |
| T1 HbA1c | **0.47 (0.11)** | **(0.21, 0.76)** | **0.41 (0.11)** | **(0.13, 0.70)** |
| Covid | -0.08 (0.08) | (-0.28, 0.14) | -0.09 (0.08) | (-0.30, 0.12) |
| CES-D | 0.07 (0.13) | (-0.20, 0.48) | -0.03 (0.14) | (-0.39, 0.34) |

*Bias corrected and accelerated bootstrapped SEs and CIs.

Standardized beta coefficients

Predictors in which CIs exclude 0 are in bold.
